# Exosome, the glass slipper for Cinderella of cancer—bladder cancer?

**DOI:** 10.1186/s12951-023-02130-8

**Published:** 2023-10-07

**Authors:** Yuanyuan Yang, Lintao Miao, Yuchao Lu, Yi Sun, Shaogang Wang

**Affiliations:** Department of Urology, Tongji Hospital, Tongji Medical College, Huazhong University of Science and Technology, Wuhan, 430030 Hubei China

**Keywords:** Exosome, Bladder cancer, Mechanism, Clinical biomarker

## Abstract

Exosomes are lipid bilayer vesicles with a diameter of 40–100 nm secreted by almost all cells. They have been found play crucial regulatory roles in various diseases. With the development of exosomes engineering technology, exosome-based drug delivery has also rapidly evolved. Bladder cancer is a worldwide disease with high morbidity and recurrence but lack of funding, so it is also called Cinderella. Some explorations have demonstrated that exosomes are important in the development, prognosis, diagnosis and drug delivery of bladder cancer. With the rapid development of Mass spectrometry and next-generation sequencing, increasing numbers of differentially expressed molecules derived from exosomes have been found in bladder cancer. Exosomes and their contents are largely involved in bladder cancer progression, engineering of these exosomes with the targeted genes improves their potential for drug delivery of bladder cancer. Furthermore, exosomes and their contents are relate to many characteristics of bladder cancer. Herein, we briefly search 59 researches to explore the cargoes encapsuled in exosomes of bladder cancer patients. We also summarize the biogenesis, function, expression profiles, engineering approaches and biological mechanisms of exosomes and their contents for the diagnosis, prognosis and drug delivery for bladder cancer. We aim to make it clear whether exosomes are the glass slippers of Cinderella.

**Graphical Abstract:**

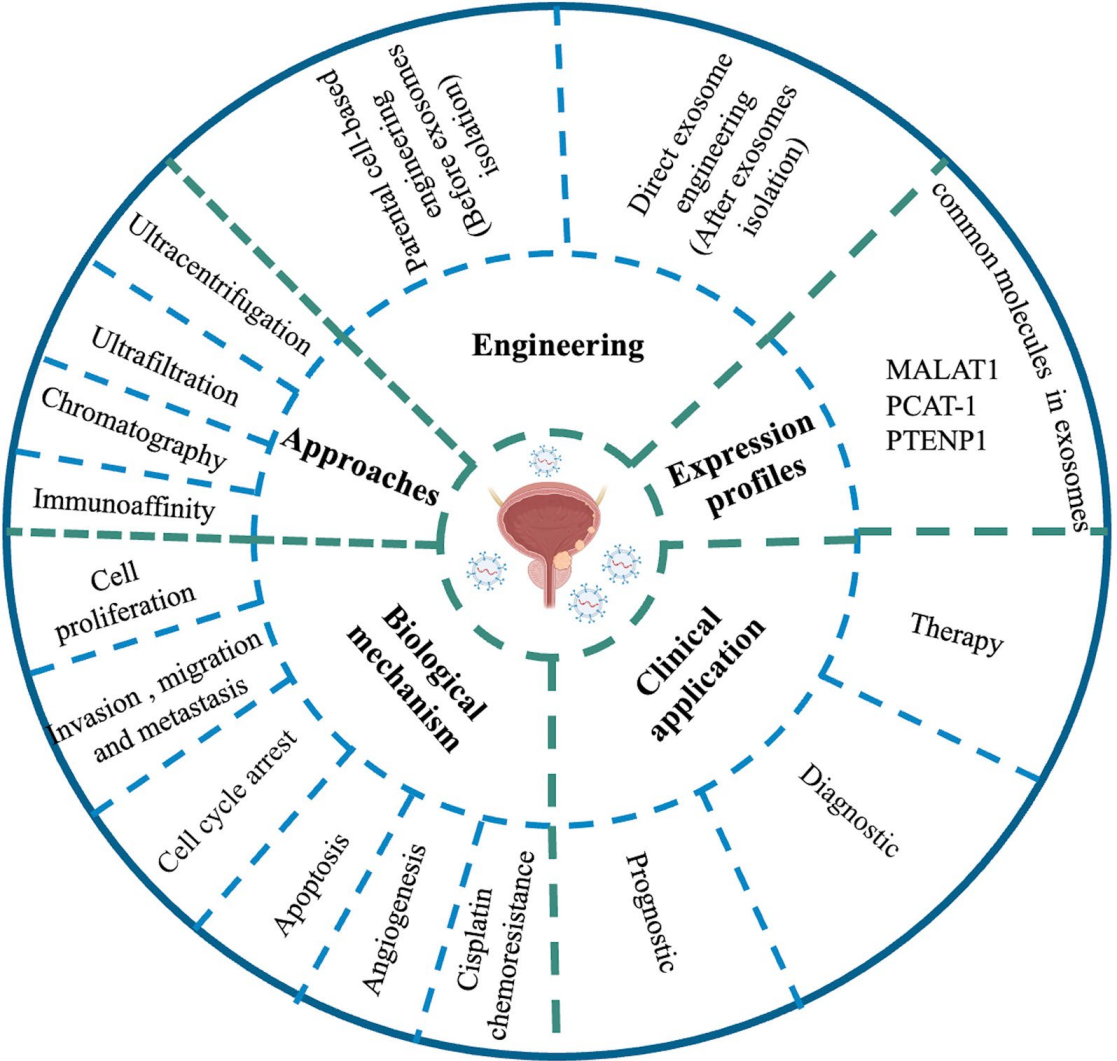

**Supplementary Information:**

The online version contains supplementary material available at 10.1186/s12951-023-02130-8.

## Background

Exosomes are spherical lipid bilayer vesicles with a diameter of 40-100 nm [1]. “Exosome” was first put forward by Trams et al. in 1981, referred as vesicles derived from plasma membrane which might play important roles in pathological and physiological function [2]. Though the concept “exosome” was widely used, ISEV 2018 guidelines suggested that it should be replaced by the term “small Extracellular Vesicles (sEVs)” [3]. For better distinction, we still refer it as “exosome” in this review. As a subtype of extracellular vesicles, exosomes distinguish themselves from microvesicles (MVs) and apoptotic bodies based on their biogenesis, size, contents and functions [4].The cargoes of exosomes include nucleic acids, lipids, cytokines and proteins [4]. Exosomes are immunogenic and can protect their contents from lysosomal degradation [5]. Exosomes have been found to play important roles in the occurrence and development of a variety of diseases through the cargoes they wrapped. More and more studies have been conducted to explore the possibility of exosomes as a treatment to cure a variety of diseases [6].

Bladder cancer is the 4th most common male cancer and 9th most common female malignancy, however, the clinical outcomes remained stagnant because of the lack of research funding. So, bladder cancer is also called Cinderella [7]. As a result, there are many unanswered questions associated with bladder cancer and needed to be explored. Recently, increasing studies have shown that exosomes play important roles in the pathological and physiology process of bladder cancer [8]. Exosomes can be used as liquid biopsy markers for diagnosis or prognosis of bladder cancer [9]. Furthermore, exosomes have been proposed as therapies for bladder cancer because they could be used for drug delivery [9]. In this review, we summarize the characteristics of exosomes and applications of engineered exosomes for drugs delivery in diseases, focused on the profiles, functions and clinical applications of exosomes in bladder cancer, we wonder whether exosomes can be the glass slippers of bladder cancer.

### Biogenesis of exosomes

The biogenesis of exosomes is intensely regulated by many cell-specific receptors and signaling pathways [10]. The first step of exosome biogenesis is the fusion of endocytic vesicles and then form early endosomes (EE) [11]. There are two pathways for EEs, one way is called “recycling endosomes”, in which EEs can return the cargoes involved in them to the plasma membrane. Or EEs can change into “late endosomes”, which also called multivesicular bodies (MVBs) through Rab5. Multivesicular bodies and late endosomes are a subset of endosomal compartments rich in intraluminal vesicles (ILVs) [12]. ILVs are originated in the inward budding of endosomal membranes, and first discovered by Pan BT et al. in mature reticulocytes [13].

The sorting of cargoes wrapped in ILVs is highly regulated by many specific molecules. Endosomal-sorting complex required for transport (ESCRT) machinery is the main mechanism mediating ubiquitinated proteins sorted into ILVs. ESCRT apparatus are consisted of four complexes, ESCRT-0, ESCRT-I, ESCRT-II, and ESCRT-III [14]. ESCRT-0 can recognize mono-ubiquitinated proteins via HRS heterodimer which is a cytosolic protein related to Clathrin. Calthrin is responsible for encountering the ubiquitinated proteins [15]. Then, the combination of ESCRT-I, ESCRT-II and ESCRT-0, binding the ubiquitinated substrates more tightly [16]. ESCRT-III finally helps to release the complex into endosome [17]. If the cargoes are not de-ubiquitinated by de-ubiquitinating enzymes (DUBs), the ILVs containing these cargoes will be targeted to fuse with the lysosome for degradation [18].

How are the un-ubiquitinated cargoes sorted into ILVs? As we know, Alix is a marker protein of exosomes and it can bind to ESCRT-III and send un-ubiquitinated molecules [19]. The ESCRT-independent pathway mainly happens in melanosomes with the help of Pmel17 and Tetraspanin CD63 [20].

Carolina Villarroya-Beltri et al. found that the specific motif in non-coding RNA decides whether it will be sorted into ILVs or not. Heterogeneous nuclear ribonucleo protein(hnRNP) is a ubiquitously expressed RNA-binding protein. Sumoylated hnRNP can recognize EXOmotifs of EXOmiRNAs and load them into ILVs. Then hnRNP can interact with cytoskeletal components to help transporting RNA to exosomes [21].

**Fig. 1:**
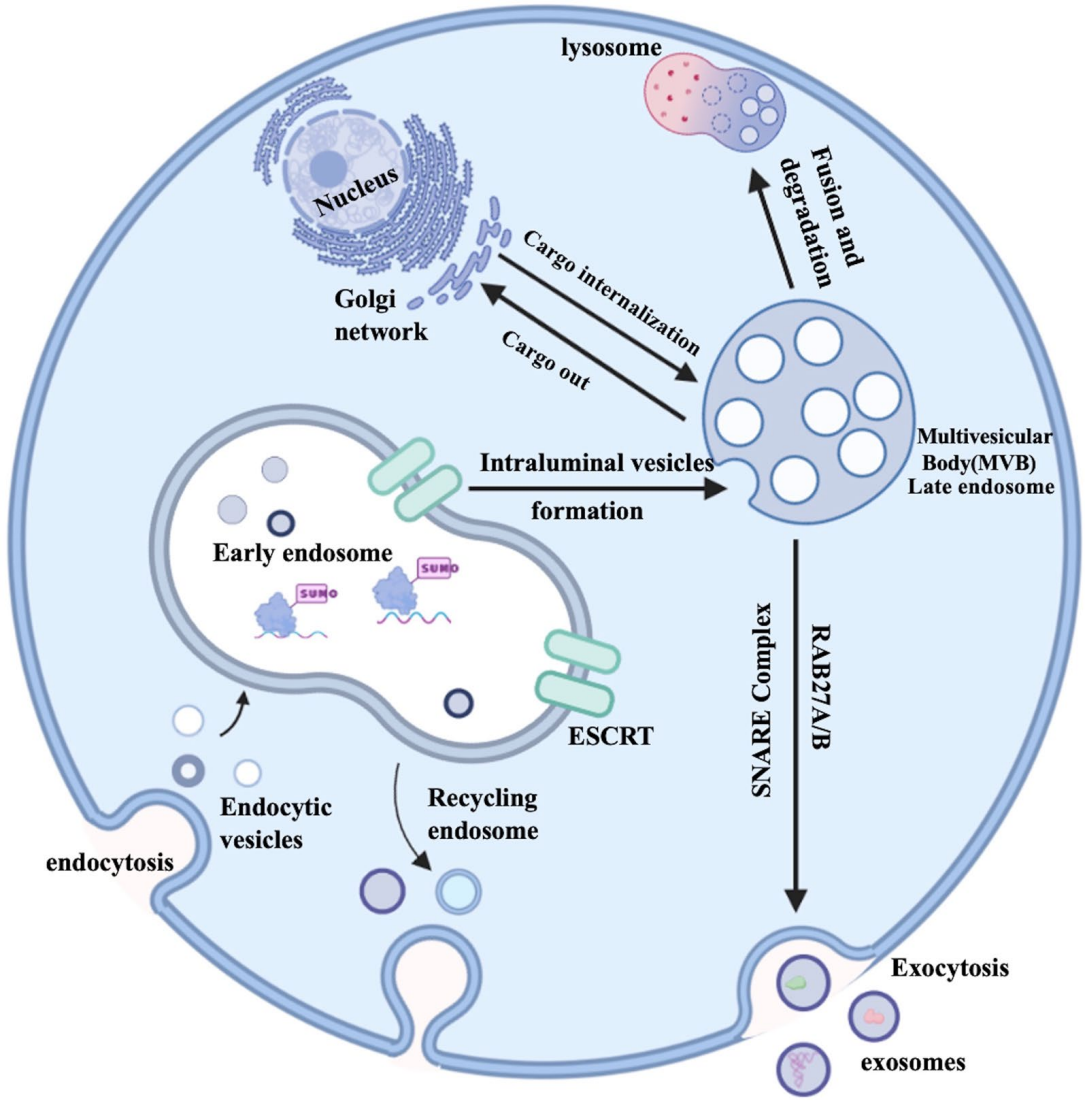
Exosomes biogenesis. In the endosomal system, endocytic vesicles fused with each other to form early endosomes (EE). There were many cargoes sequestered in EE. On one hand, the ubiquitinated proteins in EE could be sorted into intraluminal vesicles (ILVs) via ESCRT machinery. ILVs were formed through inward budding of the membrane with selected cargoes. While RNA-binding proteins heterogeneous nuclear ribonucleoprotein (hnRNP) could recognize the EXOmotifs of miRNAs and help them sorted into ILVs. On the other hand, some cargoes could be returned to the plasma membrane, called recycling endosomes. In addition, cargoes could also originate from trans-Golgi network and cytoplasm. These ILVs constituted the late endosomes /multivesicular body (LE/MVBs). The ubiquitinated targeted ILVs could be degraded within lysosome or rescue by DUBs. MVBs could also be transferred to the cell periphery via Rab27A/B. Finally, SNARE complex could help MVBs dock and fuse with plasma membrane to release ILVs into the extracellular space as exosomes

Finally, MVBs undergo two intracellular destination either fusion with lysosomes or they can move toward the plasma membrane and release ILVs to extracellular space as exosomes [22]. MVBs transferred to cell periphery are induced by Rab27A/B [23], then soluble N-ethylmaleimide(NEM)-sensitive factor attachment protein receptor(SNARE) complex drives MVBs to dock and fuse with the plasma membrane, then exosomes are released to the extracellular space [24]. Understanding the biogenesis and release of exosomes is essential for shedding new sights on therapeutic strategies (Fig. 1).

### Function of exosomes

The functions of exosomes are associated with those of mother cells and depend on the cargoes capsuled in them, they can be derived from and transferred to many types of cells mediating the intercellular communication between cells [25]. Several studies have indicated that exosomes might play important roles in immune response and infection, tumor progression, neurodegeneration, metabolic and cardiovascular diseases and inflammatory response [26]. Though no severe immune reaction has been observed elicited by exosomes [27]. Recently, research found that exosomes derived from different sources, including immune cells, epithelial, and mesenchymal cells with cargoes could regulate both the innate and adaptive immune system of recipient cells [28]. C. J. E. Wahlund et al. found that exosomes derived from antigen-presenting cells (APCs) could induce the activation of specific T cells via p-MHC-II (major histocompatibility complex II with antigen peptide(p)) capsuled in exosomes [29]. R. Nandakumar et al. found that the nucleic acid exosomal cargo, namely DNA and miRNA of intercellular bacteria played important roles in regulating immune responses [30]. The studies focused on roles exosomes playing in cancer have increased rapidly. Many studies indicated that exosomes can influence neoplasia, tumor growth and metastasis [31]. K. Stefanius et al. found that exosomes derived from pancreatic cancer can initiate cell transformation by inducing mutations in NIH/3T3 recipient cells [32]. According to M.T.Le et al.,exosomal miR-200 derived from breast cancer cells can enhance the metastasis of breast cancer [33]. What’s more, exosomes are found important in neurodegenerative disorders mainly because of their control of misfolded protein accumulation. It has been found that a-synuclein was rich in cerebrospinal fluid of patients with Parkinson or amyotrophic lateral sclerosis [34]. What’s more, Yingkun Hu et al. demonstrated that exosomes could regulate the inflammatory response mainly through NF-κB signaling pathway [26]. In addition to participating in the pathological and physiological processes of various diseases, exosomes also have numerous applications in clinical settings including designing more effective personalized treatments [35]. Although there have been many studies conducted to explore the function of exosomes, what is the core capsuled in these exosomes that maximally affect the recipient cells remains vague.

### Approaches for exosomes studies

**Fig. 2:**
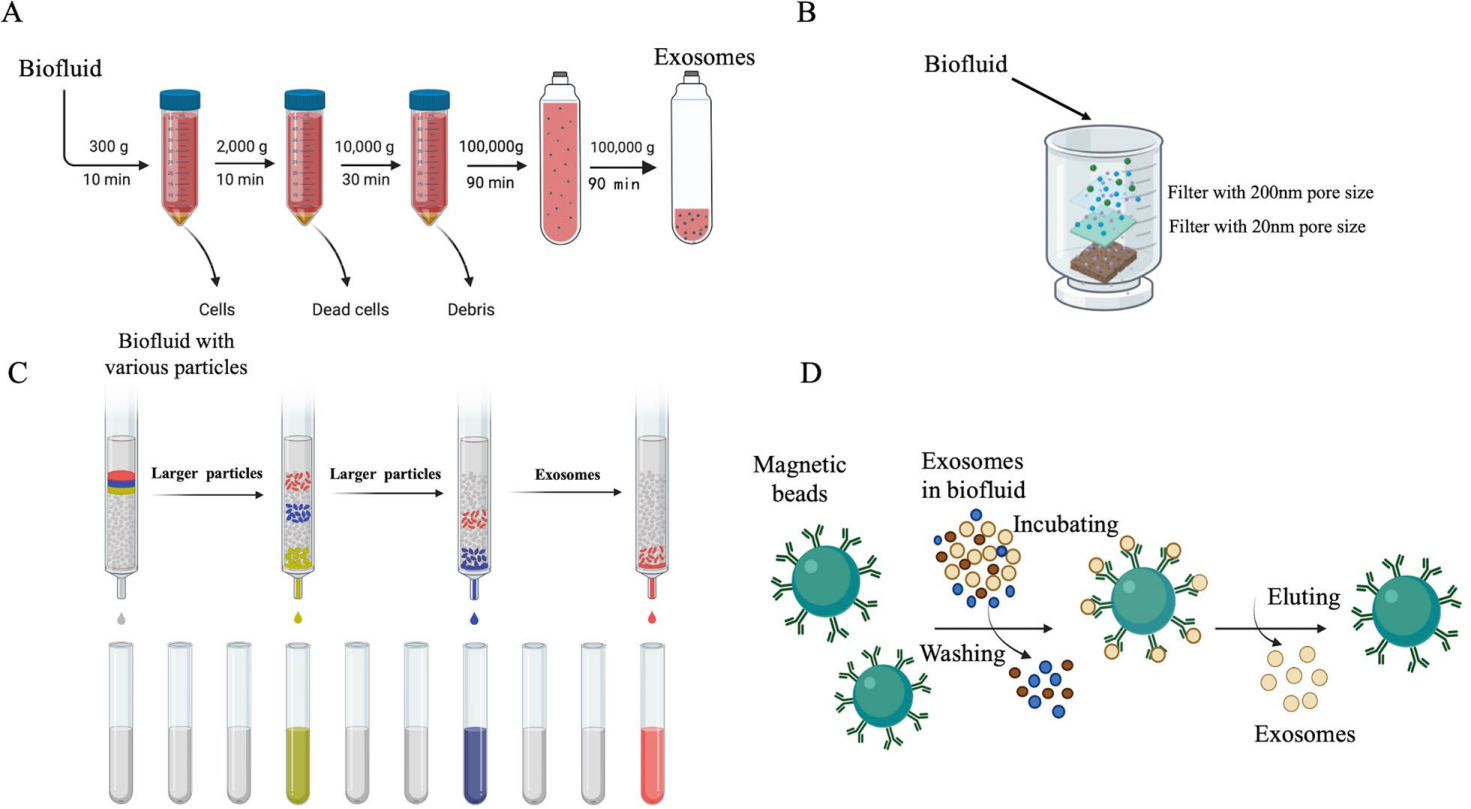
Common separation techniques. **A** Ultracentrifugation; **B** ultrafiltration; **C** Size-exclusion chromatography; **D** Immunoaffinity

Exosomes exert their effects depending on the cargo enclosed within them. For a long time, exosomes were considered merely as a mechanism for transporting cellular waste, however, with the development of mass spectrometry and next-generation sequencing, the exploration of exosomal contents has improved a lot [36]. The mostly used methods for verification of exosomes include Western blotting, NTA and TEM. Many approaches, including PCR, Western blotting, Northern blotting and ELISA are widely used to validate the cargoes capsuled in exosomes [37]. Separation of exosomes is the first step to all the exploration and utilization, many methods on exosome isolation and purification poured out in these years, namely ultracentrifugation, ultrafiltration, size-exclusion chromatography, Immunoaffinity, polymer precipitation and many commercial separation kits [38]. The large improvements in methods and experimental approaches help us learn the biogenesis and function of exosomes better (Fig. 2).

### Approaches of designing exosomes used for drug delivery

**Fig. 3:**
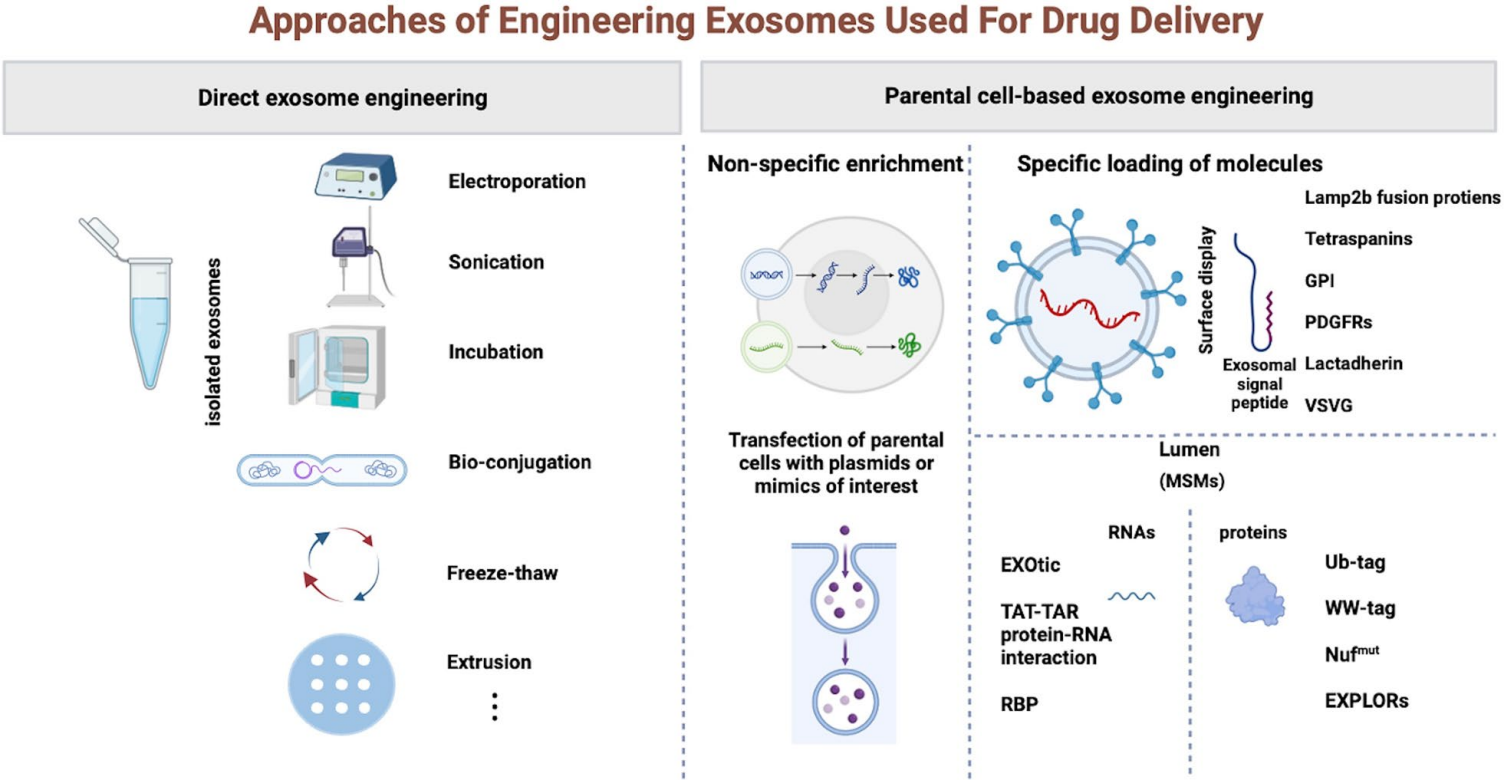
Engineering approaches of exosomes. Two main approaches of designer exosomes include parental cell-based exosome engineering and direct exosome engineering, in which the engineering procedures of parental cell-based exosomes occur before exosomes isolation from cells while direct exosome engineering occur after exosomes isolation. There are also many different methods in each class. *GPI* Glycosylphosphatidylinositol, *PDGFRs* Platelet-derived growth factor receptors, *VSVG* Vesicular stomatitis virus glycoprotein, *MSMs* Molecule sorting modules, *EXPLORs* Exosomes for protein loading via optically reversible PPIs, *EXOtic* Exosomal Transfer Into cells, *TAT-TAR* Trans-activator of transcription, Trans-activating response RNA loop, *RBP* RNA binding proteins;

The characteristics of exosomes make them a suitable platform for drug delivery [39]. Although natural exosomes have many advantages, they still have many limitations for clinical application, such as low targeting capability and a low concentration of functional molecules [40]. Engineered exosomes can effectively overcome these limitations. There are two main approaches to designing exosomes: parental cell-based exosome engineering and direct exosome engineering. The engineering procedures of the former occur before the isolation of exosomes, while the latter occurs after the isolation of exosomes. Parental cell-based exosome engineering can be divided into two classes. In the first class, non-specific way, it can be conducted through the transfection of parental cells with plasmids or mimics of interest. These procedures exclude any packaging and sorting. The other class involves specific loading of molecules, which can also be divided into two subgroups: exosomal surface display and loading into the lumen.Exosomal surface display utilizes exosomal signal peptides, including Lamp2b (lysosome-associated membrane protein 2b) fusion proteins [41], tetraspanins (CD63, CD9, CD81) [42], GPI (glycosylphosphatidylinositol) [43], PDGFRs (platelet-derived growth factor receptors) [44], lactadherin (C1C2 domain) [45], and VSVG (vesicular stomatitis virus glycoprotein) [46]. Fuse an interested protein to the signal peptide can present the protein on the exosomes’ surface. Loading of the molecules into the lumen of exosomes is based on molecule sorting modules (MSMs). Various methods with different MSMs exist, including engineered ubiquitin tags [47], WW tags [48], non-functional mutants of the HIV-I Nef protein [49], EXPLORs (exosomes for protein loading via optically reversible protein–protein interaction) for loading proteins, and EXOtic (exosomal transfer into cells) devices, TAT (Trans-activator of transcription)—TAR (Trans-activating response RNA loop) protein-RNA interaction strategies, and RNA binding modules for loading RNA into exosomes [50].Technically, direct exosome engineering is simpler compared to methods based on parental cells. In this approach, electroporation, sonication, incubation, bio-conjugation, freeze–thaw, and extrusion can be applied directly to design exosomes after their isolation from cells [51] (Fig. 3). The emergence and application of increasingly numerous and advanced engineered methods provide better tools and prospects for exosome-based drug delivery.

### Bladder cancer(Cinderella)

Bladder cancer is the 4th most common cancer in male and 9th in female. The prevalence and incidence keep increasing worldwide. However, the clinical outcomes have stayed static for 25 years for the small investment in bladder cancer research, therefore, bladder cancer is also known as “Cinderella”, often neglected though important [7]. There are three main pathological types of bladder cancer of which bladder urothelial carcinoma (BUC) accounting for 90% [52]. BUC can be composed of muscle-invasive BCa (MIBC) and non-muscle-invasive BCa (NMIBC) and NMIBC accounts for approximately 75% [53]. The treatments of bladder cancer were often endoscopic resection and adjuvant intravesical therapy and patients with advanced disease were treated with immunotherapy. Though the treatments for BCa have improved a lot through years, postoperative recurrence and distant metastasis are still severe, making it of great importance to explore the potential ways for treatment, early diagnosis, prognosis and prevention [54, 55].

### Researches of exosomes in BCa

A full review was conducted with Web of science, PubMed and Embase to search reports with the key words (“exosomes” or “extracellular vesicles”) and (“bladder cancer” or “bladder urothelial carcinoma” or “bladder neoplasm” or “bladder tumor”) for 10 years since January 2013-March 2023 Additional file 1. The studies finally involved in this review are listed in Additional file 2: Table S1. Research associated with exosomes and their contents involved in bladder cancer has increased annually. Collectively, recent studies validate exosomes derived from bladder cancer cells, or biofluid of bladder cancer patients can wrap up mRNAs, miRNAs, lncRNAs, proteins and bacteria which are crucial in the formation and metastasis of bladder cancer [56, 57].

Increasing methods have been conducted to explore the contents of exosomes. RNA sequencing (RNA-seq), microarray, 16S metagenomic sequencing and Mass Spectrometry are widely used for identification and quantification of exosomes. Western Blotting, Reverse transcription polymerase chain reaction (RT-PCR), and Enzyme linked immunosorbent assay (ELISA) are main approaches used to further verify the contents of exosomes.

To explore the function and application of exosomes more comprehensively, conveniently and efficiently, numerous exosomes-associated public databases have been established, including EVmiRNA, ExoRBase, ExoCarta, EV-TRACK, MiRandola and so on. For example, ExoRBase contains the information of exosomal circRNA, lncRNA and mRNA from human serum samples. EV-miRNA provides organ- and disease-associated miRNA annotations [58–66]. The characterization of other databases is listed in Table 1.

**Table 1:** Database for exosomes research

Database	URL	Function	Ref./PMID
EVmiRNA	http://bioinfo.life.hust.edu.cn/EVmiRNA/	Database contains miRNA expression profiles in EVs from 17diseases	30335161
ExoRBase	http://www.exorbase.org/	Database is a repository of circular RNA (circRNA), long non-coding RNA (lncRNA) and messenger RNA (mRNA) derived from human blood exosomes	34918744
Exocarta	https://www.exocarta.org/	Database provides the contents that were identified in exosomes in multiple organisms	26434508
EV-TRACK	http://evtrack.org/	Database contains the methodological parameters of EVs related research	28245209
EMBL-EBI QuickGO	https://www.ebi.ac.uk/QuickGO/	Database provides annotation for exosomal proteins	34697638
Vesiclepedia	http://www.microvesicles.org/	Database contains proteins, mRNA, miRNA, lipid, apoptotic blebs and microparticles	26861301
Urinary exosome protein database	http://hpcwebapps.cit.nih.gov/ESBL/Database/Exosome/	Database contains exosomal proteins from urine of healthy volunteers	15326289
ExRNA Atlas	http://exrna-atlas.org/exat/	Database includes miRNA derived from biofluids of human and mouse	30668638
MiRnadola	http://mirandola.iit.cnr.it	Database provides comprehensive manual classification of various types of extracellular circulating non-coding RNA	29036351

*URL* uniform resource locator

### The profiles of exosomal cargoes in bladder cancer

Many novel dysregulated exosomal cargoes have been found in bladder cancer cell lines and biofluid of bladder cancer patients, demonstrating that exosomes play important roles in bladder cancer development and progression. From Additional file 2: Table S1, we found Joanne L et al. presented the first proteomics analysis of exosomes derived from bladder cancer cell lines in 2010, they reported 353 high quality identifications of which 72 proteins were not found by other human exosome studies before, what’s more, authors found that basigin 5T4 and galectin-3 were confirmed positive in exosomes derived from urine of bladder cancer patients, indicating they might play important roles in bladder cancer formation [67]. Dennis et al. found 58 significantly different exosomal proteins derived from bladder cancer cells with or without the metastatic process, indicating exosomes could affect the metastasis and progression of bladder cancer [68]. Microarray showed urinary exosomal miR-375 and miR-146a could be used as biomarkers for high-grade and low-grade bladder cancer [69]. In another study, next generation sequencing revealed that HOTAIR and four additional lncRNAs, including HYMAI, LINC00477, LOC100506688 and OTX2-AS1 enriched in the exosomes of UBC patients, suggesting that UE-derived lncRNA could be served as biomarkers and therapeutic targets [70]. In addition, secondary bioinformatic analyses based on Gene Expression Omnibus (GEO), the Cancer Genome Atlas (TCGA) and exosome-related databases were used to identify differentially expressed exosomal protein, mRNAs and non-coding RNAs. Nitu Kumari et al. found that exosomal catanin, PAK1, CDC42 and NF2 were overexpressed in bladder cancer patients via Exocarta database and verified them in the urine samples of bladder cancer patients [71]. Bioinformatic analysis of the tissues of bladder cancer patients constructed a panel of five urinary exosomal mRNAs, then exosomes derived from urine samples were used to validate the ROC of the panel, indicating the panel a potential diagnosis of bladder cancer [70]. RNA-seq, or Mass spectrometry data analysis, paired t tests or non-parametric Mann–whitney U tests are conducted to analyse differences between groups in microarray. Fold change ≥ 2.0 is treated as significantly different and the false discovery rate (FDR) is recommended to be < 0.05. For RT-PCR or Western blotting of exosomes, an external reference is usually used instead of internal reference.

### Biological functions of exosomes in bladder cancer

#### Exosomes regulate the hallmarks of bladder cancer

**Fig. 4:**
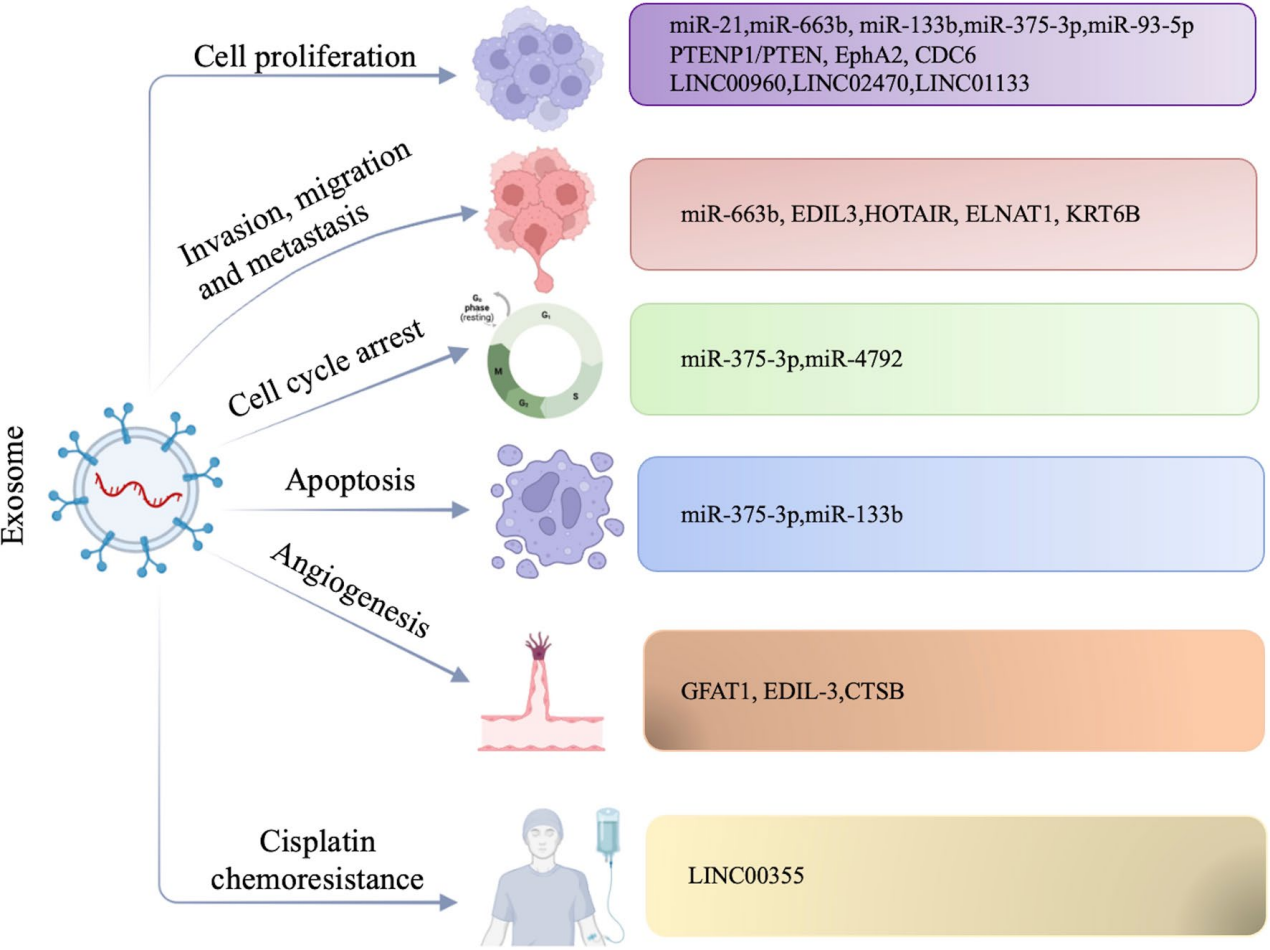
The signaling pathways involved in exosomes regulating bladder cancer progression. Exosomes and their contents can regulate cell proliferation, cell cycle, invasion and migration, metastasis, angiogenesis and cisplatin chemoresistance in bladder cancer. The main signaling pathways involved in these processes including Wnt/β-catenin pathway, PI3K/AKT pathway, STAT3 pathway and NF-κB signaling pathway

**Fig. 5:**
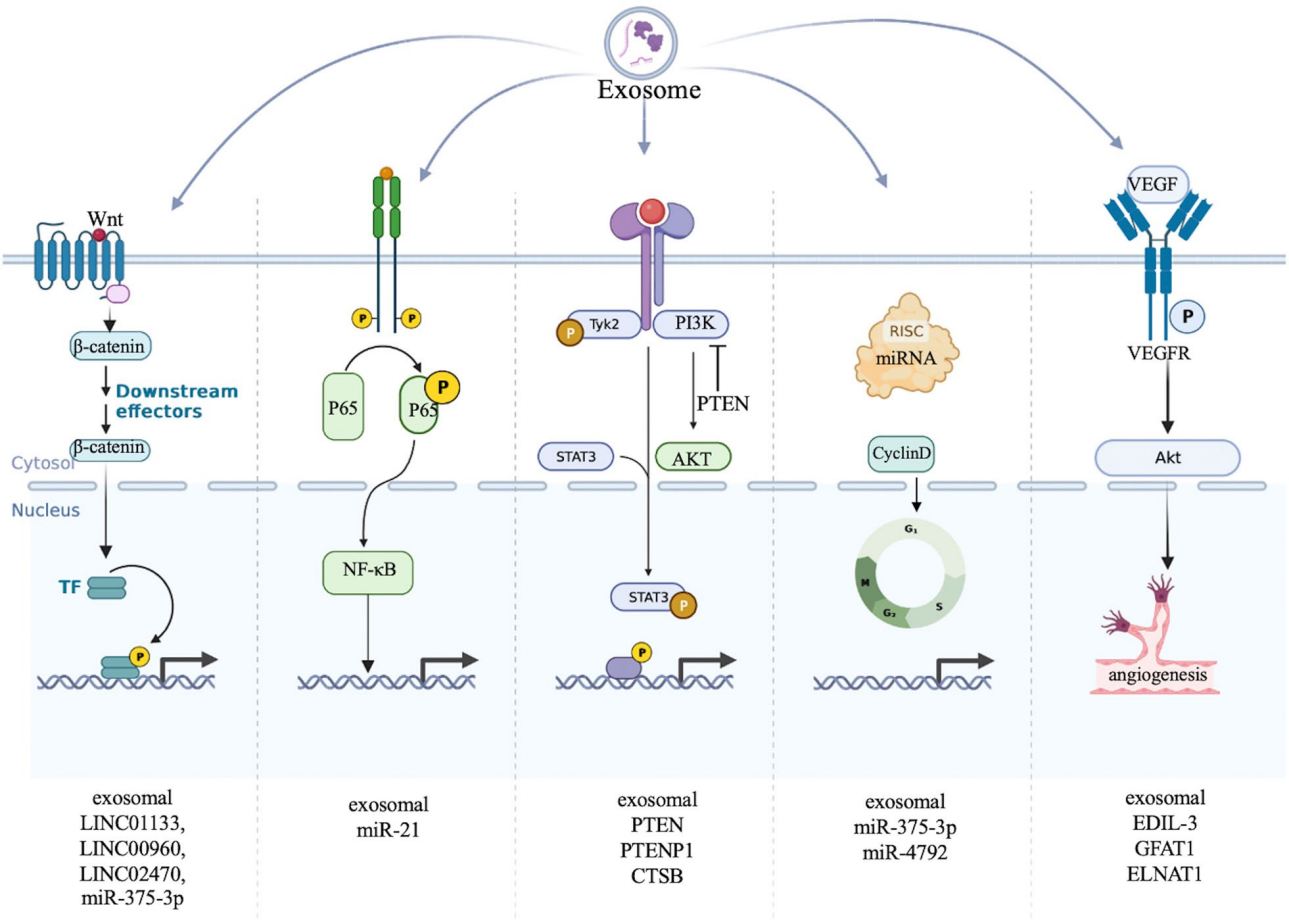
The relationship between exosomes and bladder cancer. Exosomes and their contents regulate cell proliferation, apoptosis, invasion, migration and metastasis, angiogenesis, and cisplatin chemoresistance in bladder cancer

Proliferative signaling, Growth suppressors, Cell death, Replicative immortality, Angiogenesis and Invasion and metastasis are important hallmarks of bladder cancer [72]. Here, we summarize the exosomes involved in the progression of bladder cancer to explore the association between exosomes and the hallmark features of cancer (Fig. 4). There are more and more studies indicated that exosomes could be involved in cell proliferation, apoptosis, invasion, migration, metastasis, angiogenesis and cisplatin chemoresistance of bladder cancer. We summarize the main signaling pathways involved in these processes in Fig. 5.

#### Cell proliferation

Bladder cancer can sustain proliferative states through activating cell proliferation signaling pathways [55]. Normal cells derived exosomes could regulate NF2 to inhibit tumor growth and progression of bladder cancer [71]. The PI3K/AKT/NF-κB/STAT3 signaling pathway is an important regulatory pathway. FAN LIN et al. found that exosomal miR-21 derived from bladder cancer cells could promote M2 phenotypic polarization through inhibiting phosphatase and tensin homolog activation of PI3K/AKT pathway, and finally lead to cancer progression [73]. Phosphatase and tensin homologue (PTEN) is a negative regulator of PI3K/AKT pathway. Rui Zheng et al. found that exosomes derived from normal cells transferred PTENP1 to bladder cancer cells, then exosomal PTENP1 acted as a miR-17 decoy to regulate PTEN, suppressing bladder cancer progression [74]. Consistent with the results, Shu -Cheng Liu et al. revealed BMSC-derived exosomal PTENP1 suppressed the bladder cancer by upregulating the expression of SCARA5, making it a potential target for bladder cancer therapy [75]. Exosomes derived from MB49, a kind of mouse bladder cancer cells, induced macrophage M2 polarization via down-regulation of PTEN and activation of AKT/STAT3/6 signaling [76]. MiR-663b generated from exosomes of bladder cancer cells could act as a tumor promoter via targeting Ets2-repressor factor [77]. Exosomal miR-133b could suppress bladder cancer proliferation by upregulating dual-specificity protein phosphatase1(DUSP1) [78]. Cheng Shuo Huang et al. presumed that exosome-derived LINC00960 and LINC02470 from high-grade bladder cancer cells promote the malignant progression by upregulating β-catenin signaling, Notch signaling, and Smad2/3 signaling [79]. Exosomal miR-375-3p and LINC01133 were also found to be a suppressor of bladder cancer and could inhibit proliferation and metastasis via Wnt/β-catenin pathway [80, 81]. What’s more, exosomal miR-93-5p suppressed BTG2 expression and promoted bladder cancer cells progression, exosomal EphA2 promoted the invasion and migration of bladder cancer cells, exosomal CDC6 effectively repressed the malignant process of bladder cancer cells [82–84].

Additionally, dysregulated of cell cycle regulators played important roles in bladder cancer cell growth and progression [85].C-MYC and Cyclin D1 are two key genes regulating the cell growth [56]. As reported by Qi Li, exosomal miR-375-3p could block the expression of Cyclin D1 and c-Myc and then inhibited cell growth [80]. Jian-Hong Wu et al. also provided the first evidence that the exosome-mediated delivery of miR-4792 could down-regulate c-Myc, inhibiting aerobic glycolysis [86].

#### Apoptosis

Apoptosis is one of the major mechanisms resulting in controlled cell death which can be controlled by cancer cells. Many tumor cells can avoid apoptosis, thus they can multiplicate infinitely [87]. Qi Li et al. found that exosomal miR-375-3p suppressed bladder cancer growth through promoting apoptosis in BC cells [80]. Bladder cancer cell-derived exosomes could inhibit tumor cell apoptosis via activating Akt and ERK pathways [88]. Chia-Hao Wu et al. demonstrated that tumor-derived extracellular vesicles (TEVs) could promote malignant transformation of predisposed cells by inhibiting pro-apoptotic signals [89]. According to Xiaoxiao Cai et al., exosomal miR-133b could induce apoptosis in BC cells [78].

#### Invasion and metastasis

The invasion of tumor cells into lymphatic and blood vessels is important for the metastasis of solid tumor to distant organs [90]. While epithelial-mesenchymal transition (EMT) plays an important role in the invasion and metastasis process, for which epithelial cells lose their cell polarity and cell–cell adhesion [91]. Dennis et al. found the exosomal proteins derived from bladder cancer cells with or without metastasis were significantly different, indicating the important roles these proteins might play in the metastasis process [68]. Carla et al. revealed that exosomal EDIL3 derived from bladder cancer could activate epidermal growth factor receptor signaling which induced cell migration [92]. CA Franzen et al. demonstrated that exosomes derived from bladder cancer cell were able to induce the expression of several mesenchymal markers in recipient urothelial cells [93]. Claudia et al. showed that IncRNA HOX transcript antisense RNA(HOTAIR) was increased in exosomes derived from the serum of bladder cancer patients, loss of this lncRNA in UBC cells altered expression of epithelial-to-mesenchyme (EMT) [70]. EV-mediated ELNAT1 was proved to promote lymphangiogenesis and LN metastasis in bladder cancer via UBC9/SOX18 regulatory axis, EV-mediated ELNAT1 was also correlated with a poor prognosis [94]. Consistent with these results, Changhao Chen et al. declared that bladder cancer cell-derived exosome-mediated lymphangiogenesis promoted LN metastasis in bladder cancer through a VEGF-C-independent manner [95]. MicroRNA(miR)-663b was found increased in plasma from patients with bladder cancer (BC), while it could promote epithelial-mesenchymal transition via targeted Ets2-repressor factor [78]. KRT6B, a molecule significantly related to epithelial-mesenchymal transition and immune mechanisms, was detected elevated in bladder cancer-derived exosomes, indicating its crucial role in the invasion and metastasis of bladder cancer process [96].

#### Angiogenesis

The growth and progression of tumor are highly relied on the nutrients and oxygen supplied by angiogenesis. Without angiogenesis, the size of tumor will only be limited to 200 μm [97]. Vascular endothelial growth factor (VEGF) is one of the most potent inducers of angiogenesis [98]. Exosomal GFAT1 derived from bladder cancer was reported to promote tumor angiogenesis by inducing HBP-related metabolic reprogramming and SerRS O-GlcNAcylation in endothelial cells, this may shed light on novel targets for bladder cancer antiangiogenetic therapy [99]. As illustrated by Carla J et al., exosomes isolated from high grade bladder cancer cells could promote angiogenesis and migration of bladder cancer cells. Exosomal EDIL-3 was one of the proteins that activated epidermal growth factor receptor signaling, inducing bladder cancer cell migration [92]. According to Xinyuan Li, cathepsin B (CTSB) was upregulated in exosomes derived from serum of bladder cancer patients, directly ingesting EV-CTSB prominently activated TPX2-mediated phosphorylation of the AURKA-PI3K-AKT axis, increased VEGFA expression, finally promoted angiogenesis [100].

#### Cisplatin chemoresistance

Cisplatin resistance is a problem for bladder cancer although bladder cancer is relatively sensitive to chemotherapy. Previous studies have indicated that exosomes can promote chemotherapy resistance [101]. Consistent with these results, Guangyue Luo found that exosomal LINC00355 derived from CAFs promoted the cisplatin chemoresistance of bladder cancer via the miR-34b-5p/ABCB1 axis [102].

#### Clinical significance of exosome in Bca

**Fig. 6:**
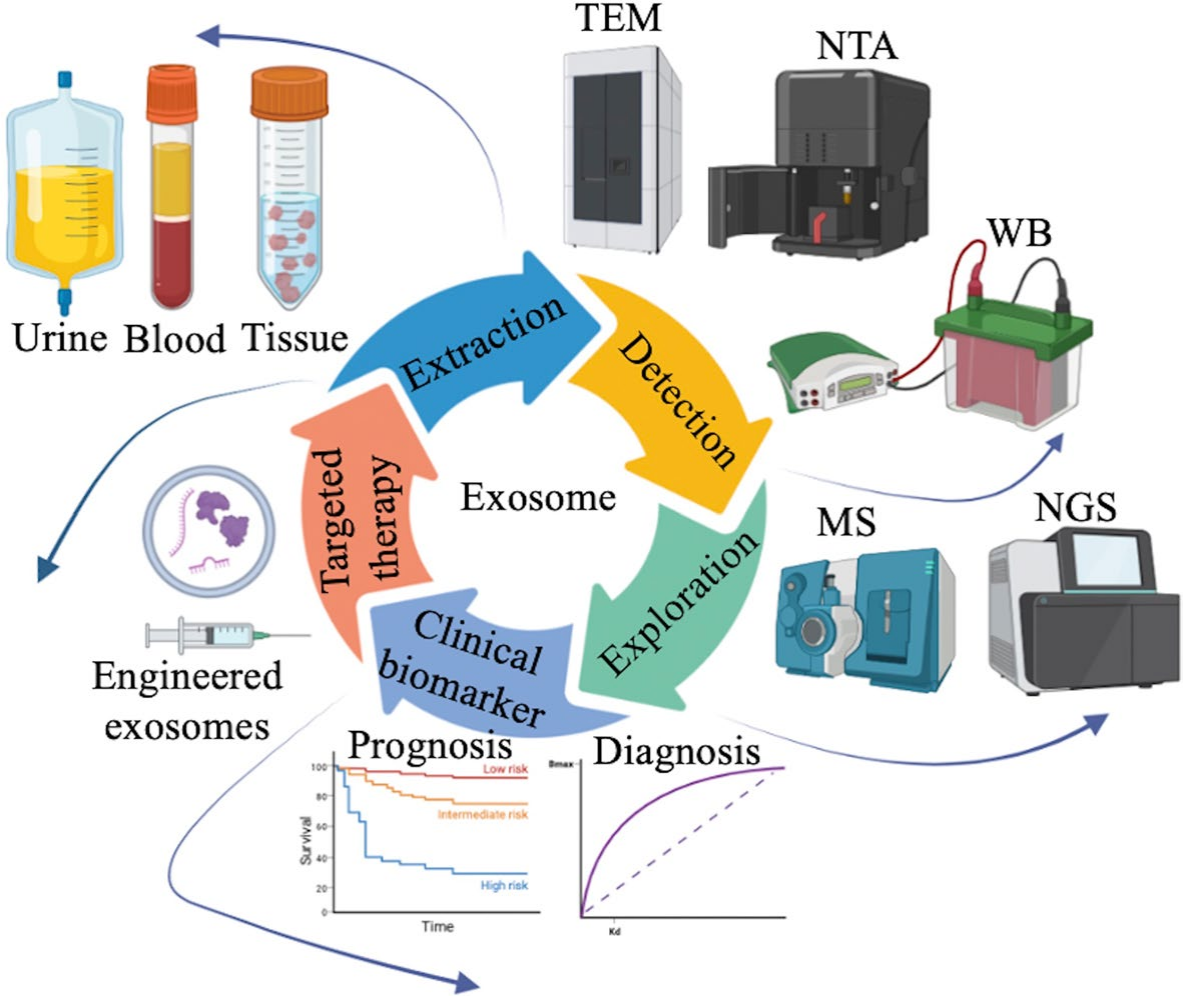
Clinical applications of exosomes in bladder cancer. Exosomes and their contents can be used as biomarkers for prognosis and diagnosis in bladder cancer. They also have the potential to become targeted therapy for bladder cancer

Bladder cancer is the second most common urology malignancy worldwide [103]. The high mortality makes it important to promote its early diagnosis and prognosis. Currently, the gold standard of the diagnosis in bladder cancer is cystoscopic examination of bladder and histological evaluation of the bladder tissue [104]. However, it is an invasive examination. Urine cytology is another common method for bladder cancer diagnosis, however its low sensitivity for low-grade tumors prevents it from widely used [105]. Exosomes are membrane-bound vesicles that most cells release into body fluids and they have been treated as mediators of tumor progression over past decades [106]. What’s more, exosomes are stable and they can protect their cargoes from degradation by enzymes [107]. Therefore, many studies have focused on the clinical applications of exosomes (Fig. 6). Exosomes with the potential of diagnostic, prognostic value of bladder cancer are listed in Table 2. As mentioned before, exosomes play key roles in bladder cancer, promoting the release of exosomes or inhibiting the secretion of exosomes might be an effective strategy for inhibiting the progression of bladder Cancer [108]. What’s more, exosomes can be designed to be loaded with exogenous RNAs and proteins for targeted therapy [109]. Engineered exosomes have been widely applied in bladder cancer, Liu et al. found Exo-miR-138-5p engineered from adipose derived mesenchymal stem cells(ADSCs) could penetrate tumor tissues and suppress the growth of xenograft tumors, what’s more, Mesenchymal stem cells-derived exosomal microRNA-139-5p restrained tumorigenesis in bladder cancer [110, 111].

**Table 2:** Exosomes for clinical management of BCa

Country	Age		Molecules	Sensitivity	Specificity	AUC	BCa
Iran	BCa:55.84 Healthy:57.4		ANRIL	46.67%	87.5%	0.7229	T1/T2
			PCAT-1	43.33%	87.5%	0.7292	
China	/		TERC	78.65%	77.8%	0.836	BCa/Healthy
Egypt	BCa: 59.5 ± 3.2		miR-96-5p	80.4%	78.4%	0.85	BC patients/Healthy
			miR-183-5p	91.8%	81.6%	0.83	
			miR-96-5p &miR-183-5p	88.2%	87.8%	0.87	
Egypt	/		Serum exosomes	82.4%	100%	0.97	BC patients/Healthy
			Urine exosomes	92.6%	83.3%	0.82	
China	Training cohort	Validation cohort	mRNA:KLHDC7B CASP14 PRSS1 lncRNA:MIR205HG GAS5	mRNA:71.9%	95.2%	0.88	BC patients/Healthy
	HCs: 45.7 ± 14.0	HCs: 47.4 ± 11.3		lncRNA:67.1%	87.1%	0.842	
	BCa: 61.4 ± 10.5	BCa: 64.8 ± 12.5		Total:88.5%	83.3%	0.924	
Japan	/		CEACAM	81.82%	97.87%	0.907	BC patients/Healthy
South Korea	BCa: 66.77 ± 11.56		Alpha-2-macroglobulin	93.3%	34.8%	0.809	BC patients/Healthy
	Healthy donors: 60.35 ± 7.40						
China	/		miR-93-5p	74.1%	90.2%	0.838	BC patients/Healthy
			miR-516-5p	72.9%	89.9%	0.79	
Japan	BCa: 72.8 ± 10.6		SLC2A1	0.64	0.75	0.7	BC patients/Healthy
			GPRC5A	0.54	0.72	0.64	
			KRT17	0.58	0.58	0.64	
South Korea	BCa: 66.77 ± 11.56		Alpha-2-macroglobulin	93.3%	34.8%	0.64	BC patients/Healthy
	Healthy donors: 60.35 ± 7.40						
Iran	BCa: 62.67 ± 11.96		TUG-1	76.67%	77.78%	0.78	BC patients/Healthy
	Healthy donors: 57.4 ± 5.7						
Japan	/		EphA2	61.1%	97.2%	0.79	BC patients/Healthy
China	/		H19	74.07%	78.08%	0.851	BC patients/Healthy
China	/		CA9	85.18%	83.15%	0.837	BC patients/Healthy
China	BCa: 68.08 ± 10.61		MYBL2,TK1,UBE2CKRT7,S100A2	88.89%	54.13%	0.8402	BC patients/Healthy
	Healthy donors: 69.89 ± 11.31						
Iran	BCa: 55.42 ± 155.55		UCA1-201,UCA1-203,MALAT1and LINC00355	92%	91.7%	0.73	BC patients/Healthy
	Healthy donors: 68 ± 13.56						
Iran	BCa: 61.28 ± 13.01		MAGE-B4	71.7%	66.7%	0.67	BC patients/Healthy
	Healthy donors: 64.42 ± 15.53						
China	/		MALAT1,PCAT-1 and SPRY4-IT1	72.1%	84.6%	0.844	BC patients/Healthy
China	/		UBC1,PCAT-1 and SNHG16	85%	78%	0.857	BC patients/Healthy
China	BCa: 67.0 ± 9.8		PTENP1	65.4%	84.2%	0.743	BC patients/Healthy
	Healthy donors: 66.2 ± 10.7						

Exosomes are significantly related to characteristics of bladder cancer. Fathia et al. observed that urine and serum exosome level is correlated with the tumor stages, indicating it can be used as biomarker for prognosis and diagnosis [108]. The contents wrapped in exosomes have been found to be involved in the clinical applications of bladder cancer. a2M (alpha-2-macroglobulin) has been reported to be upregulated in the urine exosomes of bladder cancer patients [112]. Moreover, a three exosomal lncRNA panel (RMRP, UCA1 and MALAT1) are elevated in bladder cancer, and is correlated with the tumor stage of bladder cancer [113]. Furthermore, the exosomal proteins derived from bladder cancer urine and healthy controls are significantly different, indicating their potential as a noninvasive biomarker [114]. Similarly, urine exosomal NMP22 is upregulated in bladder cancer than normal samples [115]. Chenchen et al. demonstrated that exosomal TERC is significantly upregulated in the urine of bladder cancer patients, what’s more, it has a tight correlation with tumor grade [116]. Exosomal EDIL-3 has been shown to be overexpressed in urine samples of bladder cancer patients and its levels are associated with pathologic grade [92]. Similarly, exosomal miR-375 is overexpressed in bladder cancer, and its levels are correlated with high-grade tumor. In contrast, miR-146a is downregulated in bladder cancer, and its expression levels are significantly correlated with low-grade tumor [69]. Sophie et al. revealed that exosomal miR-146b-5p and miR-155-5p derived from urine of bladder cancer patients have a positive correlation with muscle invasion of tumor [117]. According to Hao Lin et al., the expression of exosomal miR-93-5p and miR-516a-5p is higher in bladder cancer, and the level of exosomal miR-93-5p is associated with muscle invasion of tumor [82]. The expression levels of exosomal KLHDC7B, CASP14, PRSS1, MIR205HG and GAS5 have been found increased in bladder cancer urine samples, furthermore, the expression of these five molecules are significantly related to tumor stage, grade and hematuria degree [118]. In addition, exosomal TUG-1 is detectable in bladder cancer urine and serum at an early stage [119]. Exosomal BCYRN1 has been reported to be associated with lymph node metastasis of bladder cancer, and, higher expression of BCYRN1 represented poorer prognosis [120]. Alexandru et al. indicated that exosomal miR-4508 and piR-has-5936 have a tight association of risk class and tumor grade, while miR-4508 has a downward trend as the risk class increased, piR-has-5936 has a upward trend as the risk class increased [121]. Dong hyeon Lee and Xunian Zhou both found that the unique somatic variants of exoDNA are positively correlated with bladder cancer [122, 123]. In addition, label-free optic redox ratio of exosomes can also tell bladder cancer patients from normal controls [124].

There have been many studies focusing on exosomes treated as diagnostic biomarker for bladder cancer. The area under the receiver operating characteristic (ROC) curve (AUC) of exosomal CEACAM1 is 0.907 [125]. The AUC for exosomal miR-96-5p is 0.87, with a sensitivity of 82.4% and a specificity of 91.8% [126]. The AUC of combined RMRP, UCA1 and MALAT1 is 0.875, with the sensitivity of 80% and specificity of 81.4%, respectively [113]. The AUC of combined exosomal UCAI-201, UCAI-203, MALAT1 and LINC00355 is 0.96, with a sensitivity of 92% and a specificity of 91.7%, respectively [127]. The AUC for exosomal CA9 is 0.837, with a sensitivity of 85.18% and a specificity of 83.15%, respectively [128]. The AUC of exosomal TERC is 0.836, with the sensitivity of 78.65% and specificity of 77.78%, respectively The AUC of combined exosomal KLHDC7B, CASP14, PRSS1, MIR205HG and GAS5 is 0.924 [118]. The AUC of exosomal ANRIL is 0.7229, with a sensitivity of 46.67% and specificity of 87.5%, respectively [129].

Exosomes can also be used to predict the prognostic of bladder cancer. We found that upregulated exosomal H19, BCYRN1, periostin and miR-10b-5p were reported to predict poor overall survival (OS) [120, 121, 130, 131], while downregulated of exosomal TALDO1, miR-185-5p and miR-106a-5p were reported to predict poor OS [119, 121]. Cheng-shuo huang et al. revealed that the expression of exosomal LINC00960 and LINC02470 can be used as prognostic surveillance [79]. In addition, patients with high exosomal PCAT-1, UBC1, SNHG16 were reported to have a lower recurrence-free survival [132]. Similarly, two studies revealed that higher expression of exosomal miR-451a with miR-486-5p and MALAT1, PCAT1 are associated with poorer recurrence-free survival [133, 134]. In addition to these published studies, we searched the registered clinical trials website and found that SunYat-Sen Memorial Hospital has been conducting a prospective, multicenter cohort study in blaader cancer to explore the predictive value of exosomal ELNAT1 for lymphatic metastasis of bladder cancer (Additional file 1: Fig S1).

## Discussion

Bladder cancer is a worldwide disease with high morbidity and recurrence, however, there are not many studies explored on bladder cancer for the lack of funding, so it is also called “Cinderella” [7]. The mechanism and progression of bladder cancer still remain vague. What’s more, a noninvasive and accurate diagnosis or prognosis biomarker and engineered exosomes for drug delivery of bladder cancer is urgently needed. As we outlined above, exosomes and their contents are deeply involved in the formation and metastasis of bladder cancer, they can also be used as the liquid biomarker for bladder cancer. Does that mean exosome is the glass slipper of Cinderella? This question still needs further explorations.

Exosomes are spherical lipid bilayer vesicles with a diameter of 40-100 nm, they can be secreted from most cells through a period of processes [135], the contents wrapped into exosomes are sorted through ESCRT-dependent pathway or ESCRT-independent pathway [136]. The exosomes can protect their contents from degradation by RNase. The isolation and purification methods for exosomes have improved a lot over the past decades, In addition to the methods described above, combined application of those methods, such as combined ultracentrifugation and ultrafiltration can lead to the higher purity and quality of exosomes [137]. Furthermore, more and more Isolation Kits have been invented.

The cargoes wrapped in exosomes include almost all kinds of RNA, proteins, lipids and so on, they play crucial roles in the progression and metastasis of bladder cancer, they can also be used for diagnosis or prognosis in bladder cancer. The studies over the past 10 years share some common exosomal contents including MALAT1, PCAT-1 and PTENP1. Many studies have demonstrated that these three molecules play key roles in bladder cancer and can be used as accurate biomarker for bladder cancer [113, 127, 129, 132, 133]. The phosphatase and tensin homologue (PTEN) is an essential tumor suppressor [138]. It is reported to be pivotal to regulate the receptor tyrosine kinase (RTK) PI-3 kinase (PI3K)/Akt pathway [139]. PTENP1, the pseudogene of PTEN, is a novel modulator of PTEN expression [140]. The relative expression of PTEN and PTENP1 change according to the variable stages and histological grades of different tumors [141–143]. Prostate cancer associated transcript-1(PCAT-1) is an oncogenic lncRNA, high expression of PCAT-1 is associated with poor overall survival of cancer. It is also involved in *Wnt/β*-catenin-signaling pathway and participates in the cancer cell proliferation, apoptosis, invasion and metastasis [144].

Metastasis associated lung adenocarcinoma transcript 1(MALAT1) is a ubiquitous lncRNA in mammals, it is widely explored in cancer and crucial for the regulation of cancer-related pathways. MALAT1 can modulate many chief tumourigenesis pathways including MAPK/ERK, PI3K/AKT, β-catenin/Wnt, Hippo, VEGF, YAP signaling pathways, etc. [145]. What’s more, MALAT1 is reported to correlate with poor OS, RFS, DFS in various cancers [146]. However, MALAT1 also plays a key role in many other diseases like diabetes and neurologic disorders, which make MALAT1 not an ideal tumor biomarker [147, 148]. Combined some other molecules might make the detection more accurate.

In addition to the contents wrapped in exosomes, the properties of the exosomes themselves are also worth exploring. The exosomes level derived from urine samples are significantly correlated with the tumor grade and stage [108]. Jaena Park et al. found the label-free optical redox ratio of exosomes can be used for diagnosis for bladder cancer [124]. What’s more, engineered exosomes have been widely used for targeted delivery of drugs in bladder cancer, the approaches of engineered exosomes include parental cell-based exosome engineering and direct exosome engineering, Exo-miR-138-5p engineered from adipose derived mesenchymal stem cells(ADSCs) and Mesenchymal stem cells-derived exosomal microRNA-139-5p have been found restrain the growth of bladder cancer.

## Conclusion

Exosomes are spherical lipid bilayer vesicles with a diameter of 40–100 nm, the contents wrapped into exosomes are sorted through ESCRT-dependent pathway or ESCRT-independent pathway. Engineered exosomes have been used for targeted delivery of drugs in many diseases. They have been found to play crucial roles in bladder cancer progression and immigration, they can also be noninvasive biomarkers for prognosis or diagnosis of bladder cancer. Exosomal MALAT1, PCAT-1 and PTENP1 have been found in many studies focused on the link between exosomes and bladder cancer, indicating these three molecules participate in the progression of bladder cancer in depth. What’s more, engineered exosomes have been widely found to play important roles in bladder cancer. Exosomes seem to be the glass slippers of Cinderella, although it still needs further exploration whether the shoes fit well.

### Supplementary Information

**Additional file 1.** Literature search flowchart.

**Additional file 2: Table S1**. Overview of exosomes and their contents identified in bladder cancer.

## Data Availability

Not applicable.
