# Supplementary figures and images for: Exosome, the glass slipper for Cinderella of cancer—bladder cancer?
Source: J Nanobiotechnology. 2023 Oct 7;21:368. doi: 10.1186/s12951-023-02130-8 (PMC10560442; doi:10.1186/s12951-023-02130-8)

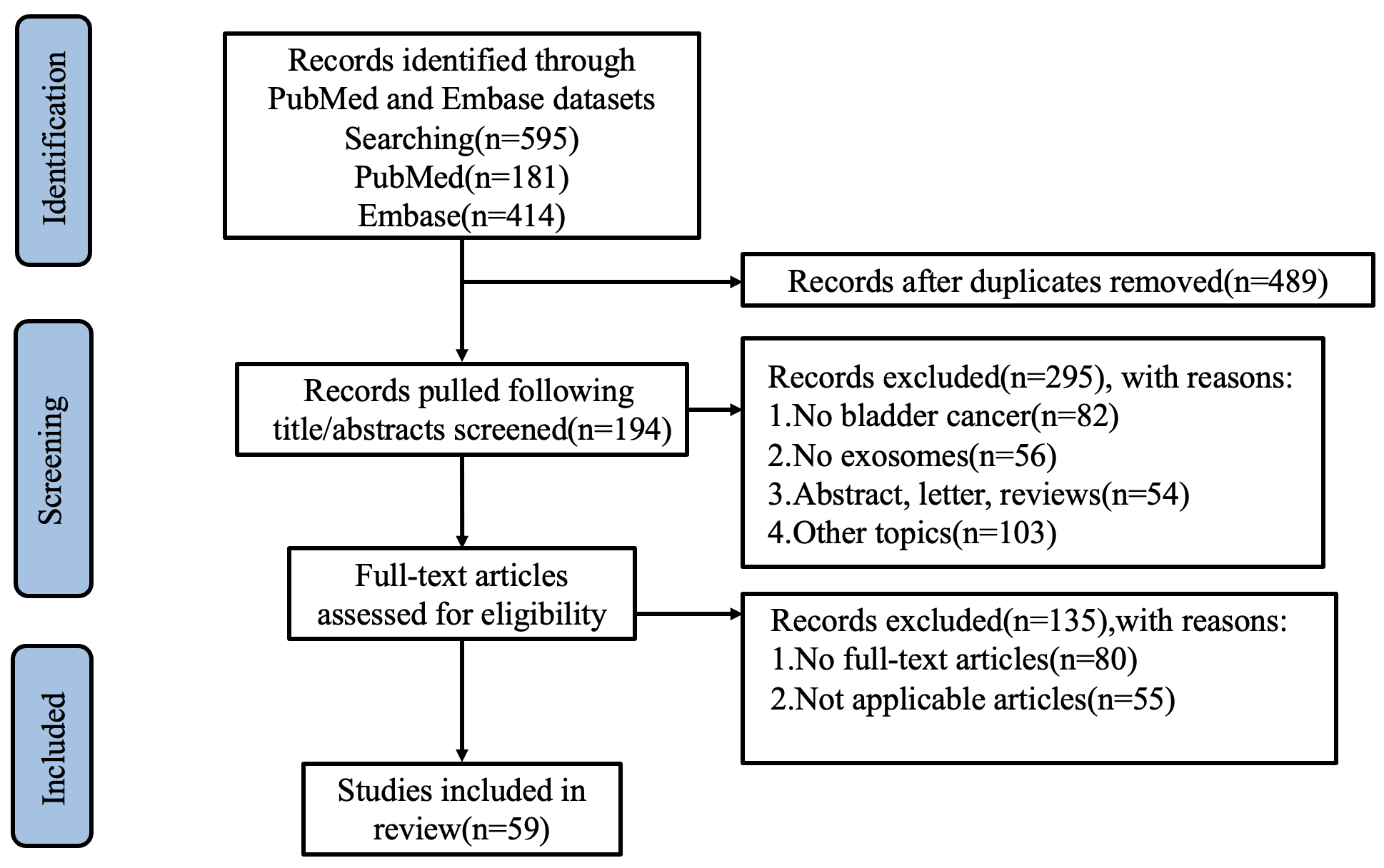

Supplement: Supplementary file 1 — Additional file 1. Literature search flowchart. [file 12951_2023_2130_MOESM1_ESM.png]
