# Supplementary material for: Exosome, the glass slipper for Cinderella of cancer—bladder cancer?
Source: J Nanobiotechnology. 2023 Oct 7;21:368. doi: 10.1186/s12951-023-02130-8 (PMC10560442; doi:10.1186/s12951-023-02130-8)
Supplement: Supplementary file 2 — Additional file 2: Table S1. Overview of exosomes and their contents identified in bladder cancer. [file 12951_2023_2130_MOESM2_ESM.docx]

**TableS1. Overview of exosomes and their contents identified in bladder cancer.**

| Authors | Year | Sample | EVs isolation  Methods | Detection  Methods | Methods used to find the molecules | Dysregulated  Molecules | Validated  Methods | Main  Outcomes | Ref/  PMID |
| --- | --- | --- | --- | --- | --- | --- | --- | --- | --- |
| *Joanne L. Welton* et al. | 2010 | HT1376 cells line | Serial centrifugation | / | LC-MALDI-TOF/TOF MS | Basigin  Galectin-3  Trophoblast glycoprotein(5T4) | WB | Authors found 353 high quality identifications in exosomes derived from HT1376 cells, of which basigin, 5T4 and galectin-3 were confirmed positive on urinary exosomes from a bladder cancer patient. | [70] |
| Lin Yang et al. | 2013 | Human bladder cancer T24 cell | Serial centrifugation | TEM  NTA  WB | / | / | / | This study demonstrated that bladder cancer cell derived exosomes inhibited tumor cell apoptosis, which activating AKT and ERK pathway, suggesting that tumor-derived exosomes are involved in bladder cancer progression. | [94] |
| *Dennis Kjølhede Jeppesen* et al. | 2014 | Human bladder carcinoma cell line T24,SLT4 and FL3 | Differential centrifugation | NTA  TEM  WB | LC-MS/MS | 58 significantly different proteins | WB | This study identified several protein changes in the membrane and lumen of exosomes from bladder cancer cells related to increased metastatic propensity. Several of the proteins with increased abundance are related to EMT. | [71] |
| Carla J.Beckham et al. | 2014 | Conditioned medium of BC cell lines(5637,TCC-SUP,T24,SV-HUC,HUVECs) and urine of BCa patients | Serial Centrifugation | EM  WB | LC-MS/MS | EDIL-3 | WB | Exosomes derived from the urine of patients with bladder cancer contains bioactive molecules such as EDIL-3. Identifying these components and their associated oncogenic pathways could lead to novel therapeutic targets and treatment strategies. | [98] |
| CA Franzen et al. | 2015 | Two MIBC cell lines | Differential centrifugation | / | / | exosomes | / | This study provided a first glimpse into the role of exosomes in bladder cancer progression, as well as recurrence. This also provided a platform for elucidating the mechanism in which exosomes are inducing EMT in urothelial cells, as well as insight into determining predictive biomarkers. | [99] |
| *Nitu Kumari* et al. | 2015 | Urine of bladder cancer patients | Differential centrifugation | / | Exocarta database | Alpha-catenin  Beta-catenin  PAK1  CDC42  NF2 | WB | This preliminary analysis showed the presence of exosomal proteins alpha-catenin, beta-catenin, PAK1, CDC42 and inferred protein NF2 in the urine to be of importance in the biology of the tumor. | [74] |
| Zoraida Andreuet et al. | 2016 | Urine of 34 BCa patients | Serial ultracentrifugation | NTA  TEM | Agilent miRNA microarray  /Mass spectrometry | miR-375  miR-146a  ApoB | qPCR  /WB | This study indicated miR-375 as a biomarker for high-grade bladder cancer while 146a as low-grade. ApoB was also a clear marker for malignancy. | [72] |
| Claudia Berrondoo et al. | 2016 | urine of BCa patients,  UBC cell lines | Serial Centrifugation | EM  WB | Illumina HiSeq2500 | HOTAIR  HYMAI  LINC00477  LOC100506688  OTX2-AS1 | qPCR | This study found that HOTAIR and four additional lncRNAs enriched in UBC patient UEs. These data, suggest that UE-derived lncRNA may potentially serve as biomarkers and therapeutic targets. | [73] |
| *ChristopherR.Silvers* et al. | 2016 | TCC-SUP MIBC cell lines | Differential centrifugation | TEM  NTA  WB | Mass spectrometry | periostin | WB | This study indicated that periostin is a component of bladder cancer cells associated with poor clinical outcome, and EVs can transfer oncogenic molecules such as periostin to affect the tumor environment and promote cancer progression. | [136] |
| Sophie  Baumgart  et al. | 2017 | Supernatant  of UBC with different degrees of invasiveness | Total Exosomes Isolation from Cell medium Kit(Life Technologies) | EM  NTA  WB | Agilent microarray  analysis | miR-30a-3p  miR-99a-5p  miR-137-3p  miR-141-3p  miR-205-5p | qPCR | Authors showed that exosomes from invasive UBC cells are characterized by a specific miRNA signature. | [156] |
| *ChristopherR.Silvers* et al. | 2017 | TCC-SUP and SV-HUC cell lines | Differential centrifugation | TEM  NTA  WB | Mass spectrometry | Transaldolase(TALDO1) | WB | This study found that exosomal transaldolase. Was of higher expression in MIBC patients and pointed the way to future investigation. | [138] |
| Fathia Elsharkawi et al. | 2018 | Urine and serum samples of 70 bladder cancer Egyptian patients from stages T0-T3 and 12 healthy control people | ExoQuant TM overall exosome capture and quantification assay Kit | / | / | exosomes | ELISA | Urine and serum exosomes are recommended as non-invasive stable and sensitive biomarkers for early diagnosis of bladder cancer, also it could be used in monitoring and prognosis of tumor at different stages. | [114] |
| *Dong Hyeon Lee* et al. | 2018 | Urine samples of bladder cancer patients | Commercial exosome precipitation reagent, ExoQuick-TC(System Biosciences, Mountain View, CA) | NTA  EM  WB | Illumina NextSeq | KMT2D  ARID1A  PIK3CA  TP53  RB1 | qPCR | This study confirmed that exoDNA could successfully identify somatic mutations and CNVs of UBC, urinary exoDNA could be another. Source of liquid biopsy. | [128] |
| *Jingyun Lee* et al. | 2018 | Urine samples of bladder cancer patients and. Healthy donors | Differential centrifugation | NTA  EM  WB | LC-MS | MUC1  CEA  EPS8L2  moesin | WB | This study demonstrated that urine EV have the potential to provide an enriched source of important biological information regarding cancer status and mechanisms of progression. | [120] |
| *Jiansong Wang* et al. | 2018 | Serum samples of BCa patients and healthy donors | ExoQuick Exosome Precipitation Solution(System Biosciences,mountainite,CA) | NTA  TEM  WB | Review | H19 | qPCR | This study demonstrated that circulating exosomal H19 released from BC cells has considerable value for the diagnosis and prognosis of BC patients in clinical applications | [137] |
| *Chia-Hao Wu* et al. | 2018 | TCCSUP，T24，SV-HUC cell lines | Serial centrifugation | EM  NTA  WB | / | / | WB | This study confirmed that TEVs promote malignant transformation of predisposed Cells by inhibiting pro-apoptotic signals and activating tumor-promoting ER stress -induced unfolded. Protein response and inflammation | [95] |
| Fatemeh Yazarlou et al. | 2018 | Urine of BCa patients | Norgen’s Urine Exosome RNA Isolation Kit(Biotek Corporatio, Thorold,ON,Canada) | EM  Dynamic light scattering(DLS)  WB | Review | UCA1-201  UCA1-203  MALAT1  LINC00355 | qPCR | This study revealed that the proposed panel of lncRNAs (composed of UCA1-201,UCA1-203,MALAT1 and LINC00355) had 92% sensitivity and 91.7%specificity for diagnosis of bladder cancer from normal samples. | [133] |
| Fatemeh Yazarlou et al. | 2018 | Urine of BCa patients | Norgen’s Urine Exosome RNA Isolation Kit(Biotek Corporatio, Thorold,ON,Canada) | EM  Dynamic light scattering(DLS)  WB | Review | MAGE-B4  NMP22 | qPCR | This study revealed that urinary exosomal MAGE-B4 had 71.7% sensitivity and 66.7%specificity for diagnosis of bladder cancer from normal samples. | [106][121] |
| Yao Zhan et al. | 2018 | Urine samples of BCa patients and healthy donors | / | TEM  NTA  WB | Review | MALAT1  PCAT-1  SPRY4-IT1 | qPCR | This study revealed a panel consisting of three differently expressed lncRNAs(MALAT1,PCAT-1 and SPRY4-IT1) was established for BC diagnosis, with a specificity of 72.1%, a specificity of 84.6% and an AUC of 0.844 | [140] |
| Shujun Zhang et al. | 2018 | Serum samples of BCa patients and healthy donors | Exo-Quick solution(EXOQ5A-1;SBI system Biosciences,USA) | TEM  NTA  WB | Review | PCAT-1  UBC1  SNHG16 | qPCR | This study revealed a panel consisting of three differently expressed lncRNAs(PCAT-1,UBC1 and SNHG16) was established for BC diagnosis, with a specificity of 85%, a specificity of 78% and an AUC of 0.857 | [139] |
| Rui Zheng et al. | 2018 | Plasma samples of BCa patients and healthy donors  Normal human cell line(HEK 293A) | Exoquick exosome precipitation solution(System Biosciences) | TEM  NTA  WB | Review | PTENP1 | qPCR | This study revealed that exosomal PTENP1 was a promising novel biomarker used for the clinical detection of BC with the AUC of 0.743,and it could reduce the progression of BC, participate in normal-cell-to-bladder-cell communication during the carcinogenesis of BC. | [79] |
| Maryam  Abbastaber et al. | 2019 | Urine of BCa patients and healthy control | Norgen’s urine exosome RNA isolation kit | SEM  TEM  WB  DLS | / | PVT-1, ANRIL, PCAT-1 | qPCR | This study indicated that ANRIL and PCAT-1 could be used as potential biomarkers in bladder cancer. | [135] |
| Sophie Baumgart et al. | 2019 | Urine of 37 BCa patients | Total Exosomes Isolation from Urine Kit(Life Technologies) | EM  NTA  WB | Agilent microarray  analysis | miR-146b-5p  miR-155-5p | qPCR | A different expression was verified for miR-146b-5p (*P* = 0.004) and miR-155-5p (*P* = 0.036), which exhibited significantly higher expression in urinary EVs from patients with MIBC. | [123] |
| *FanLin* et al. | 2019 | Human bladder T24 cancer cells | Differential centrifugation | NTA  EM  WB | review | miR-21 | qPCR | This study confirmed that bladder cancer cell-secreted exosomal miR-21 activates the PI3K/AKT pathway in macrophages to promote cancer progression. | [78] |
| Xinbao Yin et al. | 2019 | Plasma samples of BCa patients and healthy donors and human BC cell lines(T24 and 5637) | ExoQuick-TCExosome Precipitation Solution(System Biosciences,Mountain View,CA,USA) | / | Review | miR-663b | qPCR | This study revealed that exosomal miR-663b was a promising potential biomarker and target for clinical detection and therapy in BC. | [82] |
| Xiaoxiao Cai et al. | 2020 | 20 serum specimens of bladder cancer patients and 20 healthy serum specimens | Total exosome isolation reagent( ThermoFisher Scientific, MA, USA) | TEM  WB | TCGA database | miR-133b | qPCR | Exosomal miR-133b could suppress tumor growth in vivo. In addition, we found that exosomal miR-133b may play a role in suppressing BC proliferation by upregulating dual-specificity protein phosphatase 1 (DUSP1). These findings may offer promise for new therapeutic directions of BC. | [83] |
| Changhao Chen et al. | 2020 | Urine and blood samples of 206 patients with BCa and 120 healthy participants | / | / | Next-generation sequencing | LNMAT2 | qPCR | This study provided evidence of a VEGF-C-independent LN metastasis mechanism in which BCa cell-secreted exosomal LNMAT2 promoted lymphangiogenesis and LN metastasis. What’s more, they found that *LNMAT2* was overexpressed in both the uri- nary-EXO and serum-EXO of BCa patients, which positively cor- related with both intratumoral and peritumoral lymphangiogenesis, and was clinically relevant to BCa LN metastasis. | [101] |
| Stefanie Hiltbrunner et al. | 2020 | Urine from the bladder prior to surgery, and directly from the ureters after transsection | Differential centrifugation | NTA  TEM | Mass spectrometry | TPP1  TMPRSS2  FOLR1  RALB  RAB35 | WB | In authors’ proposed model, exosomes retain a malignant memory phenotype in the bladder even after TUR-B plus NAC, emphasising the importance of radical over minor surgery to remove the source of tumour-promoting exosomes. | [157] |
| Cheng-Shuo Huang et al. | 2020 | High-grade bladder cancer cell lines(T24 and J82) and low-grade bladder cancer cell lines(TSGH-8301 and TSGH-9202) | Differential centrifugation | NTA  TEM  WB | lnCAR database | LINC00960  LINC02470 | qPCR | This study indicated that exosome-derived LINC00960 and LINC02470 from high-grade bladder cancer cells promote the malignant behaviors of recipient low-grade bladder cancer cells and induce EMT by upregulating β-catenin signaling, Notch signaling, and Smad2/3 signaling. Both lncRNAs may serve as potential liquid biomarkers for the prognostic surveillance of bladder cancer progression. | [84] |
| *Qi Li* et al. | 2020 | T24 and SV-HUC-1 Cell lines | Differential centrifugation | NTA  EM  WB | Agilent 2200 TapeStation | miR-375-3p | qPCR | This study revealed that miR-375-3p is a suppressor in BC, it could inhibit BC cell growth, decrease the migration ability, promote cell apoptosis in BC, suggesting that it can be used as a therapeutic candidate for BC in the future. | [85] |
| *Alexandru A. Sabo* et al. | 2020 | Plasma samples from BC patients and healthy controls | ExoQuick exosome precipitation solution(System Biosciences,Mountain View,CA,USA) | / | Illumina HiSeq 2500 | miR-126-3p  piR-5936  miR-4508  miR-185-5p  miR-10b-5p  miR-106a-5p | qPCR | This study indicated that miR-4508 was downregulated in high risk BC and piR-has-5936 ,miR-126-3p was upregulated. Additionally, BC cases with low expression of miR-185-5p and miR-106a-5p or high expression of miR-10b-5p showed shorter survival. | [127] |
| *Mohammad Sarfi* et al. | 2020 | Urine samples from BC patients and healthy controls | Urine exosomal RNA isolation Kit（Norgen Biotek，Thorold，Canada） | TEM  NTA  WB | Review | TUG-1 | qPCR | This study showed that TUG-1 is significantly upregulated in early stages of bladder cancer and could be a potential non-invasive urinary exosome-based biomarker. | [125] |
| Yong Xu et al. | 2020 | Urine samples of population with suspected bladder cancer | Serial centrifugation | TEM  NTA  WB | TCGA dataset | MYBL2  TK1  UBE2C  KRT7  S100A2 | qPCR | This study discovered a panel of five urinary exosomal mRNAs(MYBL2,TK1,UBE2C,KRT7 and S100A2), and found the AUC was 0.8402, which could be a potential diagnosis of bladder cancer. | [75] |
| Hongjie Yang et al. | 2020 | SV-HUC-1 and BC cell lines | Serial centrifugation | TEM  NTA  WB | Review | LINC01133 | qPCR | This study confirmed that exosomes-mediated transfer of lncRNA LINC01133 repressed BC progression via regulating the Wnt signaling pathway. | [86] |
| Changhao Chen et al. | 2021 | Urine of 5 MIBC patients and 5 healthy volunteers | Differential centrifugation | TEM  NTA  WB | TCGA database | ELNAT1 | qPCR | This study made an elucidation of the precise mechanism of EV-mediated *ELNAT1* in activating the hnRNPA1/UBC9/ SOX18 axis to induce BCa LN metastasis | [100] |
| Maria Eldh et al. | 2021 | Urinary bladder tissue from tumor-proximal sites as well as tumor distant sites in TUR-B patients with or without preoperative neoadjuvant chemotherapy prior to ensuing radical cystectomy | Differential centrifugation | TEM  NTA  WB | MS | PGK1  ALDOA  GSTP1  PKM | WB | This study found the most abundant proteins in bladder tissue-derived exosomes, regardless of site, were enriched in metabolic carcinogenesis-related pathways and are linked to poor prognosis, and there was a continuous release of malignant exosomes from transformed cells in the entire bladder despite complete downstaging. | [158] |
| Haiming Huang et al. | 2021 | Urine samples from the bladder cancer patients and healthy people | Exosomes commercial kit (Norgen Biotek Corp, Product No.47200, Canada) | NTA  TEM  WB | Illumina HiSeq X Ten | KLHDC7B  CASP14  PRSS1  MIR205HG  GAS5 | qPCR | This was the first study to illustrate the combination of exosomal mRNAs and lncRNAs for the diagnosis of BCa and authors identified five molecules as promising biomarkers for an early stage BCa diagnosis. | [124] |
| *Ziming Jiang* et al. | 2021 | Bladder cancer cells MB49 | Differential centrifugation | TEM  WB | / | exosomes | / | This study confirmed the contribution of bladder cancer derived exosomes on the establishment of immunosuppressive TME and provided a potential therapeutic target for bladder cancer treatment. | [81] |
| *Kentaro Jingushi* et al. | 2021 | Serum of BCa patients and healthy donors | Differential centrifugation | NTA | 16S metagenomic sequencing | Firmicutes | 16S rRNA gene PCR | This study indicated that Firmicutes abundance derived from EVs in the blood can reflect the local immune status of the tumor microenvironment and could be used to predict the efficacy and prognosis of UC immunotherapy. | [159] |
| *Xinyuan Li* et al. | 2021 | Peripheral blood and tumor tissues of patients with or without bladder cancer | Differential centrifugation | NTA  EM  WB | LC-MS/MS | CTSB  (cathepsin B) | WB | This study demonstrated that active CTSB is upregulated in tumor size, muscle invasion, distant metastasis, and poor prognosis. What’s more, it uncovered a novel molecular pathway by which exogenously active CTSB facilitates TPX2-mediated phosphorylation of the AURKA-PI3K-AKT axis in ECs but also illuminates its unexpected function in angiogenesis. | [106] |
| *Hao Lin* et al. | 2021 | Urine samples of bladder cancer patients and non-cancerous participants | Differential centrifugation | NTA  EM  WB | Illumina Hiseq X ten platform | miR-93-5p  miR-516a-5p | qPCR | This study revealed that urine derived exosomal miR-93-5p,miR-516a-5p could be used as a promising non-invasive tool to detect BC. In vitro experiments suggested that miR-93-5p overexpression may contribute to BC progression via suppressing BTG2 expression. | [87] |
| *Guangyue Luo* et al. | 2021 | Normal fibroblasts(NFs) and BC tumor-derived CAFs. | Differential centrifugation | NTA  EM  WB | review | LINC00355 | qPCR | This study indicated that exosomal LINC00355 derived from CAFs promotes BC cell resistance to cisplatin by regulating the miR-34b-5p/ABCB1 axis. | [108] |
| *Olaf Stromme* et al. | 2021 | Urine and serum samples of NMIBC patients and non-cancer patients（NCP） | Differential centrifugation | NTA  TEM  WB | Illumina HiSeq4000 Sequencing System | miR-451a  miR-486-5p | qPCR | This is the first study to investigate EV-containing miRNA sequencing in pre-and postsurgery BC patient samples and our findings suggest that urinary EV-contained miR-451a and miR-486-5p may be potential biomarkers for recurrence-free survival of BC patients with stage T1 disease. | [141] |
| *Xiaotian Tan* et al. | 2021 | UM-UC-3,UM-UC-5,UM-UC-6,UM-UC-9 and HUC-1 cell lines | Differential centrifugation | NTA  TEM  WB | / | EGFR  HER2  MHC-1  EpCAM | ELISA | This study showed that the expression level of functional EV membrane proteins is stable under external stimulation, which suggests that the expression profile of the EV membrane proteins may serve as a robust and unique ” molecular fingerprint” for the immunophenotyping of cancer cell lines. | [160] |
| *Eisuke Tomiyama* et al. | 2021 | Urine samples and tissue-exudative of BCa patients and healthy donors | Differential centrifugation | NTA  TEM  WB | LC-MS/MS | HSP90  SDC1  MARCKS | SRM/MRM analysis | Proteomic analysis of urinary EVs and Te-EVs re- vealed that most of the proteins identified in Te-EVs also present in urinary EVs. The novel strategy that combined proteomic analysis of urinary EVs and Te-EVs enabled the identification of reliable uri- nary EV biomarker proteins (HSP90, SDC1, and MARCKS) for BCa detection. | [88] |
| *JinWen* et al. | 2021 | Urine samples of BCa patients and healthy controls | Urinary exosome isolation solution(Hope Tech Biotechnology,Guangzhou) | TEM  Flow Cytometry | Review | CA9 | q-PCR | This study revealed that urinary exosomal CA9 was abundant in the urine of bladder cancer patients with a sensitivity of 85.18% and a specificity of 83.15%, AUC of 0.837. | [134] |
| Hanhao Zheng et al. | 2021 | Urine samples of BCa patients and healthy donors | / | TEM  NTA  WB | Hiseq4000 | BCYRN1 | qPCR | This study revealed that exosomal BCYRN1 could enhance VEGF-C/VEGFR3 signaling-induced lymphatic metastasis of BCa, indicating that BCYRN1 may serve as a therapeutic target for patients with BCa. | [126] |
| Xunian Zhou et al. | 2021 | Urine and serum samples of BCa patients | Serial centrifugation | TEM  NTA  WB | Whole-exome saquencing | DNA somatic variants | / | This study demonstrated that genomic DNA could be found in exosomes from urine of individuals with BC but not in healthy samples, which could be used to identify cancer-specific mutational profiles for BC. | [129] |
| Chenchen  et al. | 2022 | Urine of 94 healthy controls, 46 patients with urinary benign lesions and 128 BCa patients. | Differential centrifugation and exosomes extraction kit(BestBio, Shanghai, China) | TEM  NTA  WB | Next generation sequencing | TERC | qPCR | This study found that TERC was significantly upregulated in urinary exosomes from BLCA patients compared with those from healthy controls (P<0:0001). Urinary exosomal TERC showed higher sensitivity (78.65%) and accuracy (77.78%) than existing indicators | [122] |
| Amal S.EI-Shal et al. | 2022 | Urine samples of 51 patients with BC,21 patients with benign urinary bladder lesions and in 24 normal individuals | miRCURY^TM^ Exosome Isolation Kit(Qiagen, Hilden Germany) | TEM  NTA  WB | Reviews | miR-96-5p  miR-183-5p | qPCR | This study found that urine miR-96-5p and miR-183-5p are promising tumor biomarkers of BC diagnosis; particularly, when they combined with each other or with urinary cytology. | [132] |
| Ko lgami et al. | 2022 | Urine samples from 31 UCB patients and 18 noncancerous patients | Differential centrifugation | NTA  TEM  WB | MS/MS | CEACAM | WB | This study identified EVs could specifically express CEACAM proteins in urine and have potential for diagnostic applications. These EVs are potential targets in a new liquid biopsy test for UCB patients. | [131] |
| *Jisu Lee* et al. | 2022 | Urine samples of bladder cancer patients and healthy donors | ExoLutE Urine kit | NTA  EM  WB | Proteome Profiler. Antibody. array | Alpha-2-macroglobulin | WB | This study demonstrated the potential of a2M as a novel uEV biomarker for BC diagnosis through an optimized EV isolation and analysis workflow. | [118] |
| *Xinyuan Li* et al. | 2022 | HUVECs  UM-UC-3  293T cell lines  And urine samples from MIBC patients | Differential centrifugation | NTA  EM  WB | Metabolomics | GFAT1 | ELISA | This study provided a new perspective on the effect of BCa-derived sEVs-GFAT1 on metabolic reprogramming in the TME and expand understanding of HBP-induced SerRS O-GlcNAcylation in ECs on promoting tumor angiogenesis. | [105] |
| *Shu-Cheng Liu* et al. | 2022 | Human BMSC(Bone mesenchymal stem cells) cell lines | Exosome isolation reagent(Thermo Fisher Scientific,USA) | NTA  EM  WB | review | PTENP1 | qPCR | This study indicated that BMSC-derived exosomal PTENP1 suppressed the BC progression by upregulating the expression of SCARAS via sponging miR-17, offering a potential novel therapeutic target for BC therapy. | [80] |
| *Jaena Park* et al. | 2022 | Urine samples from dogs with TCC of the bladder | Differential centrifugation | NTA  TEM  WB | / | Label-free optical redox ratio | SLAM microscope imaging | This study successfully showed that the label-free optical redox ratio of uEVs，indicating relative rates of glycolysis and oxidative phosphorylation of parent cells and tissues，may act as a potential screening biomarker for bladder cancer. | [130] |
| *Tongtong Qiu* et al. | 2022 | Urine samples from BC patients and normal donors | Differential centrifugation | NTA  TEM  WB | TCGA database | RMRP  UCA1  MALAT1 | qPCR | This study indicated that a three-lncRNA panel (RMRP, UCA1 and MALAT1) has a potential value for BC diagnosis in the clinic with an average AUC of 0.875, sensitivity of 80% and specificity of 81.4%. | [119] |
| *Yang Shen* et al. | 2022 | EJ and J82 cell lines | ExoQuick(System Biosciences) exosome precipitation solution | TEM  NTA  WB | Agilent Bioanalyzer 2100 | CDC6 | qPCR | This study indicated that Hirudin was able to decrease the expression of CDC6 in bladder cancer exosomes, which effectively repressed the malignant processes of bladder cancer cells. | [89] |
| *Qiang Song* et al. | 2022 | ExoCarta database | / | / | TCGA database and GEO database | KRT6B | qPCR | This study found that KRT6B, which can be detected in bladder cancer-derived exosomes, plays an important role in the epi- thelial–mesenchymal transition and immune responses in bladder cancer. Further research will enable its potentially prognostic marker and therapeutic target for bladder cancer. | [102] |
| Jian-Hong Wu et al. | 2022 | Human Adipose-Derived Mesenchymal Stem Cell(hAMSC) | Serial centrifugation | EM  NTA  WB | Review | miR-4792 | qPCR | This study provided the first evidence that the exosome-mediated delivery of miR-4792 could downregulate FOXC1 and c-Myc, which further inhibited aerobic glycolysis and lactic acid content. | [161] |
